# Mechanistic study on the susceptibility of *Staphylococcus aureus* to common antimicrobial preservatives mediated by wall teichoic acids

**DOI:** 10.1128/aem.01023-25

**Published:** 2025-06-30

**Authors:** Xia Wu, Jiayi Wang, Ji Li, Zheng Su, Jian Zha

**Affiliations:** 1 School of Food Science and Engineering, School of Biological and Pharmaceutical Sciences, Shaanxi University of Science and Technology 664015, Xi'an, Shaanxi, China; 2 Xi’an Key Laboratory of Antiviral and Antimicrobial-Resistant Bacteria Therapeutics Research, Xi’an, Shaanxi, China; 3 School of Bioresources and Materials Engineering, Shaanxi University of Science and Technology 74618, Xi'an, Shaanxi, China; INRS Armand-Frappier Sante Biotechnologie Research Centre, Laval, Quebec, Canada

**Keywords:** *Staphylococcus aureus*, wall teichoic acid, preservative, sodium dehydroacetate, tea polyphenol, polylysine, susceptibility

## Abstract

*Staphylococcus aureus* is a common food-borne pathogen that easily develops resistance to antibiotics and antimicrobial peptides partly because of wall teichoic acids (WTAs), the peptidoglycan-anchored polymers important for cell physiology. In the food industry, there is an increasing prevalence of *S. aureus* despite the widespread use of antimicrobial preservatives, and it remains elusive how WTAs affect the susceptibility of *S. aureus* to these preservatives. In this study, we first identified that the presence of WTAs altered *S. aureus* sensitivity to tea polyphenol, sodium dehydroacetate, and ε-polylysine after screening 14 frequently used antimicrobial food preservatives. Then, via a series of genetic and biochemical analyses combined with molecular dynamics simulation, we revealed three WTA-related mechanisms adopted by *S. aureus* for self-protection against sodium dehydroacetate and ε-polylysine: (i) charge repulsion mediated by D-alanylation to reduce preservative packing around the cell; (ii) hydrogen bonding and hydrophobic interactions involving WTA backbone and glycosylation to maintain the preservatives in the WTA layer and to reduce their passage across the cell membrane; (iii) steric hindrance to reduce cell wall permeability and minimize the contact of these antimicrobials with the cell membrane. In comparison, the latter two mechanisms were involved in cell interaction with tea polyphenol. This work highlights the complex roles played by WTAs in cell response to antimicrobial preservatives and provides guidance to further study on *S. aureus* tolerance to food preservatives and better control over food safety.

**IMPORTANCE:**

*Staphylococcus aureus* is a disease-causing bacterium frequently detected in raw and packaged food that can be strongly insensitive to many bacteria-inhibiting or bacteria-killing agents. With the widespread use of antimicrobial food preservatives during food processing and packaging, there is a potential risk that these preservatives may force *S. aureus* to become less sensitive. Given that *S. aureus* tolerates many antimicrobial agents using mechanisms related to wall teichoic acids (WTAs), the negatively charged polymers that are anchored in the cell wall of this bacterium, it is necessary to evaluate whether *S. aureus* presents WTA-dependent sensitivity to antimicrobial food preservatives and how WTAs affect *S. aureus* interaction with these preservatives. Our study answered these questions for tea polyphenol, sodium dehydroacetate, and ε-polylysine and revealed three WTA-related mechanisms including charge repulsion, surface trapping, and decline in cell wall permeability. This work emphasizes the need for further control over food safety.

## INTRODUCTION

*Staphylococcus aureus* is a common pathogen that causes serious infections all over the world. This bacterium easily develops resistance to antibiotics and antimicrobial peptides/enzymes via various mechanisms, among which wall teichoic acids (WTAs) are important players (1–3). WTAs are prevalent in gram-positive bacteria with various structures and are anchored in peptidoglycan, constituting a major component of the cell wall (2). These molecules are actively involved in cell division and cell wall biosynthesis, surface adhesion and biofilm formation, invasion into mammalian cells and interaction with host immune systems, and development of pathogenicity and antigenicity (4–6).

**Fig 1:**
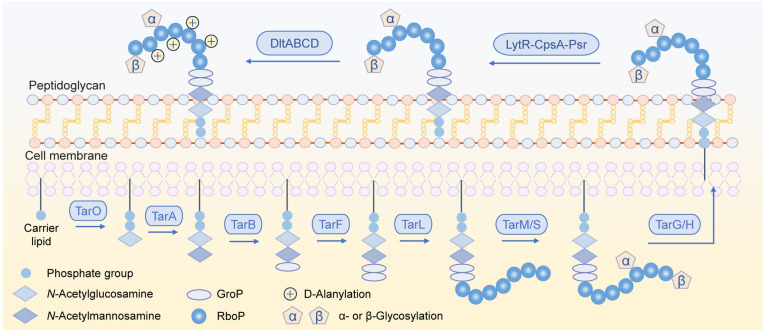
Biosynthesis of polyribitol-phosphate WTAs in *S. aureus*. The *tarM* gene is absent in strain ATCC 6538.

*S. aureus* WTAs are mostly polyribitol phosphate polymers and less commonly adopt the polyglycerol phosphate structure in certain strains (2, 7). Biosynthesis of the former type of WTAs initiates with the transfer of *N*-acetylglucosamine-1-phosphate and *N*-acetylmannosamine to a carrier lipid catalyzed by TarO and TarA, respectively, followed by the addition of two glycerol phosphate units by TarB and TarF (Fig. 1). This basic structural unit is elongated by TarL via repeated addition of ribitol phosphate units, flipped across the cell membrane by TarGH, and then translocated to peptidoglycan. These compounds can be modified at the free hydroxyl groups in the repeating unit via α-/β-glycosylation by TarM/TarS or via D-alanylation by DltABCD, thus changing the cell surface properties (1, 8). WTA biosynthesis can be regulated by transcriptional factors including Agr and MgrA as well as two-component systems GraRS and ArlRS (2, 9).

WTAs of *S. aureus* and other gram-positive bacteria mediate cell tolerance and resistance to various antimicrobials through several mechanisms. First, WTAs as charged polyelectrolytes alternate between extended and random-coiled conformations depending on environmental conditions, thereby controlling the exposure of the peptidoglycan to macromolecular antimicrobials such as bacteriolytic enzymes (10, 11). Second, the glycosylation pattern of WTAs has a great impact on phage binding and drug susceptibility (12–14). Third, WTA D-alanylation, which is the attachment of D-alanine to the hydroxyl groups and the formation of D-alanyl esters, introduces positive charges due to the dissociation of amino groups and partially neutralizes the negative charges on phosphate groups (1, 2). Such a modification results in the repulsion of positively charged antibiotics and cationic antimicrobial peptides, leading to lower sensitivity (15).

Studies on WTA-related antimicrobial resistance in bacteria have long been focusing on antibiotics, phages, and host immune systems (such as antimicrobial peptides and fatty acids) that are closely related to medical environments. In comparison, much less attention has been paid to bacterial resistance in food-processing environments. Researchers have isolated *Listeria monocytogenes* from turkey-processing plants that are resistant to Phage 100 (16), which has been approved by the US Food and Drug Administration as a biocontrol agent for food processing (17). These isolates are characterized by a lack of WTA glycosylation with *N*-acetylglucosamine or rhamnose due to mutations in the corresponding genes (16). *S. aureus* as a typical food-borne pathogen is prevalent in meat, dairy products, and ready-to-eat food, and many of these isolates are resistant to antibiotics including methicillin and vancomycin (18–22) using mechanisms related to WTA glycosylation (2, 12, 23).

During food processing, antimicrobial preservatives are usually adopted individually or in combination to prevent food spoilage by microbes, and some of these preservatives, such as sodium dehydroacetate (SD), are also widely used in cosmetics and personal care products. Currently, with very limited information, it is unclear whether repetitive exposure of *S. aureus* to these compounds will lead to lower sensitivity and whether such a process is linked to WTAs. Actually, resistance to nisin, a natural lantibiotic that is commonly supplemented in processed meat such as sausages, has already been observed in both *S. aureus* and *L. monocytogenes*, and the resistance in the latter bacterium is attributed to the absence of *N*-acetylglucosamine modification of the WTAs and lipoteichoic acids (LTAs) due to the loss-of-function mutation of the *gtcA* gene (24). For *S. aureus*, nisin resistance appears to be correlated with transcriptional regulatory systems (25–28) and transporters (26, 29), which are indirectly associated with WTAs (25) and affect cell surface properties including charge and hydrophobicity (30). As for other preservatives, however, there are no reports on resistance development in *S. aureus*.

Given the prevalence of *S. aureus* in food and its rapid establishment of resistance to antimicrobials via WTA-dependent mechanisms, it is necessary to investigate the function of WTAs in mediating *S. aureus* interaction with and vulnerability to food preservatives. Therefore, we compared in this work the susceptibility of *S. aureus* wild-type (WT) and WTA-deficient cells to a panel of antimicrobial food preservatives and identified three preservatives, i.e., SD, ε-polylysine (PL), and tea polyphenol (TP), whose growth-inhibiting activity was sensitive to the presence of WTAs. Using a series of characterization at the molecular and cellular levels, we show that WTAs as a whole affect cell susceptibility to TP, whereas WTA glycosylation and D-alanylation have a great impact on cell susceptibility to SD and PL. We also propose possible modes of interaction between *S. aureus* WTAs and these preservatives. This work emphasizes the importance of susceptibility evaluation on *S. aureus* and other bacteria, especially those isolated from food-processing procedures, to commonly used antimicrobial food preservatives, and points out further attention that is required to enhance food safety and public health.

## MATERIALS AND METHODS

### Molecular cloning and strain construction

*S. aureus* ATCC 6538 and RN4220 cells were grown in a tryptic soy broth (TSB) medium and supplemented with antibiotics when necessary. *Escherichia coli* DH5α was grown in lysogeny broth supplemented with proper antibiotics for plasmid construction.

*S. aureus* ATCC 6538 Δ*tarO* strain is a lab stock constructed previously (31). For increased *tarO* expression, the *tarO* gene was amplified from the genomic DNA of *S. aureus* ATCC 6538 using primer pair tarO-F/R (Table S1) and cloned into plasmid pLI50 between *BamH*I and *Sal*I. The plasmid was transformed into *E. coli* DH5α and then into *S. aureus* RN4220. The plasmid was then extracted and further transformed into *S. aureus* ATCC 6538 cells via electroporation, resulting in the strain with increased *tarO* expression. The same plasmid was also transformed into the Δ*tarO* strain to obtain the complementation strain. The plasmid was extracted and verified by sequencing. The original empty pLI50 plasmid was transformed into *S. aureus* ATCC 6538 wild type and Δ*tarO* cells as controls.

Gene *tarS* or *tarM* was deleted from the genome of *S. aureus* RN4220 following a reported protocol (31) with few modifications. Briefly, the two homologous flanking regions targeting *tarS* or *tarM* were amplified from the genomic DNA of *S. aureus* RN4220. The fragments were then assembled into the plasmid pUC19 and cloned into plasmid pKOR1 using in-fusion cloning. The resultant plasmids were then transformed into *S. aureus* RN4220 cells. The *tarS* or *tarM* knockouts were constructed via two‐step homologous recombination. The correct knockouts were verified by PCR amplification and sequencing. The double knockout of *tarMS* was constructed by knocking out *tarM* in the Δ*tarS* strain background. All the primers used in the strain construction are listed in Table S1.

For all the cloning work, the enzymes were purchased from Takara Bio, and kits for genomic DNA extraction, plasmid miniprep, and gene cleanup were from TIANGEN Biotech. Primer synthesis and gene sequencing were performed by Sangon Biotech.

### Measurement of MICs

The measurement of the MICs of food preservatives followed a reported protocol (32). Briefly, overnight cultures inoculated from glycerol stocks were sub-cultured into fresh TSB media and grown at 37°C and 200 rpm till the optical density at 600 nm (OD_600_) reached ~0.6. Cells were collected and diluted in fresh TSB to a final concentration of 2 × 10^5^ CFU/mL and mixed 1:1 with different concentrations (twofold serial dilutions) of food preservatives in 96-well plates to a final volume of 100 µL. Negative controls included cells in the medium mixed with the solvents that were used for the preparation of various compound solutions. The plates were kept at 37°C for 24 h. The MICs were determined as the lowest concentrations of the tested compounds that resulted in no visible sign of cell growth.

### Isolation and electrophoretic analysis of WTAs

WTAs were extracted from the wild-type and recombinant *S. aureus* strains following a well-established protocol (11). Briefly, cells were harvested when OD_600_ reached 0.6–1.0, washed with deionized (DI) water, and boiled in 4% SDS at 95°C for 1 h. After five washes with 50 mM Tris-HCl (pH 8), cells were resuspended in 20 mM Tris-HCl (pH 8) containing 0.1 mg/mL Proteinase K (Sigma‐Aldrich) and 0.5% SDS and incubated at 50°C for 4 h. The pellet was washed extensively with DI water and resuspended in 0.1 M NaOH. After 16 h of incubation with gentle shaking, the mixture was centrifuged at 13,800 g for 10 min, and the supernatant containing isolated WTAs was collected and stored at 4°C. The extracted WTAs were analyzed by polyacrylamide gel electrophoresis (PAGE) as reported (10). Samples were run in 20% gels, stained with alcian blue, and destained with DI water.

### Sensitivity testing on preservative-containing plates

Autoclaved TSB media were cooled and mixed with various concentrations of TP, SD, or PL and aliquoted into sterile petri dishes for solidification. Sub-cultures of *S. aureus* ATCC 6538 and the Δ*tarO* mutant cells were grown until OD_600_ reached ~0.6. The cultures were diluted to OD_600_ = 0.2, serially diluted, and spotted (5 µL) on TSB-agar plates containing food preservatives. After overnight incubation at 37°C, plates were photographed for visual observation.

### Cell viability assay

*S. aureus* cells after 24 h incubation with various compounds in liquid media were serially diluted. Culture dilutions were either spotted (5 µL) or spread (50–100 μL) on TSB agar plates and incubated overnight at 37°C. Colonies were counted for the calculation of cell viability.

### Scanning electron microscopy

Cells sub-cultured from overnight cultivation were grown till OD_600_ reached ~0.6. After centrifugation and washing, cells were mixed with TP (30 µg/mL), PL (0.5 mg/mL), or SD (4 mg/mL), and diluted with fresh medium to an OD_600_ of 0.2. The suspension was then cultivated for 2 h at 37°C and 200 rpm, centrifuged at 2,000 g for 5 min, and washed three times with phosphate buffered saline (PBS, pH 7.4). Sample preparation followed a reported protocol (33). Briefly, cells were fixed with 2.5% (vol/vol) glutaraldehyde at 4°C for 12 h and dehydrated with increasing concentrations of ethanol (30%–100% [vol/vol]). For each centrifugation step, cells were spun at 2,000 g for 5 min. Dried cells were coated with gold and observed under a field emission scanning electron microscope (FEI, USA).

### Quantitative reverse-transcription PCR analysis

*S. aureus* ATCC 6538 wild-type cells were grown in TSB medium at 37°C and 200 rpm when OD_600_ reached ~0.6. The culture was supplemented with TP, SD, or PL at final concentrations of 0.625 mg/mL, 8 mg/mL, or 100 mg/mL, respectively. Cells were harvested at various time points by centrifugation and kept at −80°C. RNA was isolated by TRIzol reagent (Invitrogen), treated with DNase I (Sigma-Aldrich) for genomic DNA removal, and reverse transcribed to cDNA using the High‐Capacity RNA‐to‐cDNA Kit (Applied Biosystems). Measurement of cDNA abundance was performed on a StepOne RT‐PCR machine (Applied BioSystems) using SYBR Green Master Mix (ThermoFisher Scientific) and primer pairs listed in Table S1. The gene abundance was normalized to that of the reference gene 16S rDNA.

### Isothermal titration calorimetry analysis

The isolated WTAs were neutralized with HCl, dialyzed against DI water, and lyophilized in a freeze-dryer (SCIENTZ). WTAs and food preservatives were dissolved in DI water, and isothermal titration calorimetry (ITC) was performed on a MicroCal ITC-200 (Malvern Panalytical) at 25°C. WTAs (280 µL) were loaded into the sample cell, and 2 µL aliquots of TP (2 mM, 0.6 mg/mL), SD (10 mM, 1.9 mg/mL), or PL (2 mM, 3.8 mg/mL) were injected in 19 batches with 3 min time intervals between injections, and the mixture was stirred at 500 rpm. The concentrations of WTAs were prepared as 0.2 mM, 1 mM, and 0.02 mM to determine the interactions with TP, SD, and PL, respectively. Besides, TP, SD, or PL injected into water was used as the negative control. Binding isotherms were obtained by integrating each injection peak (subtracted by that of the negative control experiment).

### Modeling of the interactions between WTAs and food preservatives

Molecular dynamics simulation was performed using the software Materials Studio 2020 and the COMPASS forcefield (34) to predict the interactions between WTAs and the food preservatives. The degrees of polymerization of WTA and ε-polylysine were set as 15 and 30, respectively (12). The Amorphous Cell module of Materials Studio was adopted to build WTA-TP, WTA-SD, and WTA-PL water boxes (6 × 6 × 6 nm^3^), with the structure optimized and energy minimized. In each of the boxes, one WTA molecule was placed together with three TP molecules, four SD molecules, or one PL molecule. Water was added to fill the boxes. The constant number, volume, temperature (NVT) ensemble was adopted for 120 ps simulation with structure relaxation. The output structures were then used as input for a 120 ps simulation employing the constant number, pressure, temperature (NPT) ensemble at 298 K and 101 kPa. The output structures were further subjected to a 100 ps simulation with 1 ps step width at 298 K and 101 kPa using the constant number, volume, energy (NVE) ensemble. Interaction of WTA with TP, SD, or PL was analyzed for various functional groups by the radial distribution function, which means the average local number density of particle A at distance *r* around the reference particle R divided by the bulk number density of particle A.

### Cytochrome c binding assay

The cytochrome c binding assay was performed based on a reported protocol (35). Briefly, the overnight culture of *S. aureus* ATCC 6538 was sub-cultured into fresh TSB medium containing 10 or 20 µg/mL of amsacrine dissolved in dimethyl sulfoxide (DMSO) and grown at 37°C and 200 rpm to OD_600_ = 0.6. Cells were then diluted 1,000-folds into fresh TSB medium containing the same concentration of amsacrine. Cells treated in the same way with DMSO and cells without any treatment were used as controls. When OD_600_ reached ~0.6, cells were collected and washed twice with 20 mM 3-morpholinopropanesulfonic acid (MOPS) buffer (pH 7) and resuspended to a calculated OD_600_ of 5. Cells were mixed 4:1 (vol/vol) with 5 g/L of cytochrome c from horse heart (dissolved in DI water) and incubated at room temperature for 10 min. The mixtures were centrifuged at 12,000 rpm for 2 min, and the supernatant was measured for absorbance at 530 nm in a microplate reader (Tecan Life Sciences), which was used for the quantification of cytochrome c concentration.

### Cell susceptibility to antibiotics and food preservatives after amsacrine treatment

Overnight cultures of *S. aureus* ATCC 6538 were sub-cultured into fresh TSB medium containing a certain concentration of amsacrine and grown to OD_600_ = 0.6. Cells were then diluted by 1,000-fold into fresh TSB media containing amsacrine (same concentration as in the first sub-culture) and various concentrations of gentamicin, neomycin, TP, SD, or PL in 96-well plates. After 24 h of incubation, the culture turbidity of cells exposed to gentamicin and neomycin was inspected for MIC determination, and the viability of cells exposed to food preservatives was determined via serial dilution and plating as described in “Cell viability assay,” above.

### Statistical methods

All experiments were performed with three biological replicates unless otherwise stated. Statistical analysis was performed using a two-tailed Student *t*-test with the software Microsoft Excel 2021, and *P* < 0.05 was regarded as statistically significant. All data are expressed as means ± standard deviations.

## RESULTS

### Screening of food preservatives that show WTA-related efficacy toward *S. aureus*

To understand whether WTAs of *S. aureus* have any impact on cell susceptibility to food preservatives, we selected 14 commonly used food preservatives and tested their MICs at pH 7.4 toward *S. aureus* and its mutant lacking WTAs via *tarO* deletion. Strain ATCC 6538 was adopted because it is one of the standard strains for antimicrobial testing (36). As shown in Table 1, while most of these compounds were equally effective toward the wild-type and the Δ*tarO* mutant, TP and SD showed twofold lower MICs, while PL had a twofold higher MIC against the *tarO* deletion strain compared with the wild-type strain. These results suggest distinct roles played by the WTA in mediating cell sensitivity to these food preservatives. It is worth mentioning that sodium benzoate, potassium sorbate, sodium lactate, and calcium propionate are effective in food preservation only in the pH range of 3–5, under which condition *S. aureus* showed impaired or no growth (Fig. S1). At a near-neutral pH, these compounds showed very high MICs with no discrimination between the wild-type and the mutant strains.

**TABLE 1:** MICs of commonly used food preservatives against *S. aureus* ATCC 6538 WT strain and the *tarO*-deficient mutant

Food preservative	MIC for:		Fold change (WT/∆*tarO*)
	WT strain	∆*tarO* strain	
Sodium benzoate	63 mg/mL	63 mg/mL	1
Potassium sorbate	75 mg/mL	75 mg/mL	1
PL	1 mg/mL	2 mg/mL	0.5
SD	15.6 mg/mL	7.8 mg/mL	2
Sodium lactate	100 mg/mL	100 mg/mL	1
Potassium metabisulfite	750 µg/mL	750 µg/mL	1
Sodium D-isoascorbate	>60 mg/mL	>60 mg/mL	1
Sodium diacetate	7.8 mg/mL	7.8 mg/mL	1
Calcium propionate	50 mg/mL	50 mg/mL	1
TPs	160 µg/mL	80 µg/mL	2
Chitosan	750 µg/mL	750 µg/mL	1
Allicin	>10 mg/mL	>10 mg/mL	1
Citral	400 µg/mL	400 µg/mL	1
Nisin	500 IU/mL	500 IU/mL	1

### Confirmation of the correlation between WTAs and cell susceptibility to food preservatives

**Fig 2:**
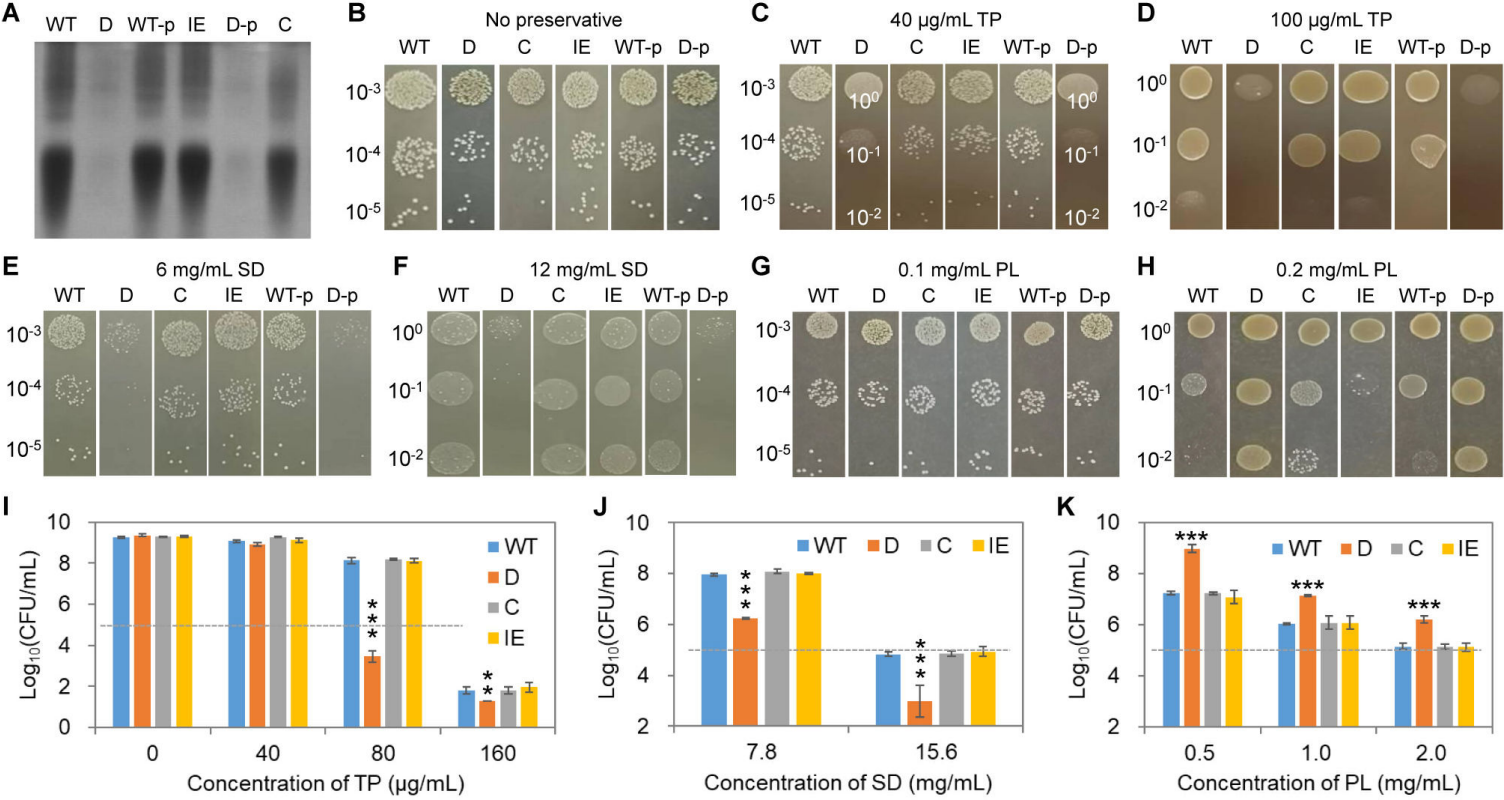
Correlation between WTAs and *S. aureus* sensitivity to food preservatives. (**A**) PAGE analysis of isolated WTAs from various recombinant strains of *S. aureus*. (**B–H**) Growth and colony formation of various strains on TSB-agar plates containing different concentrations of food preservatives representative of biological triplicates. (**I–K**) Viable cell counts of various strains cultivated in TSB media containing food preservatives. Dashed lines indicate the initial cell counts. WT, wild-type *S. aureus* ATCC 6538; D, the *tarO*-deletion strain; C, the *tarO*-complementation strain, i.e., the Δ*tarO* strain carrying the plasmid pLI50-*tarO*; IE, the strain with increased *tarO* expression, i.e., the WT strain carrying the pLI50-*tarO* plasmid; WT-p and D-p, the WT strain and the deletion strain carrying the empty plasmid pLI50, respectively. Data are presented as mean ± standard deviation of biological triplicates. **, *P* < 0.01; ***, *P* < 0.001.

To further confirm the impact of WTAs on cell susceptibility to the screened food preservatives, we constructed strains with complemented or increased *tarO* expression using episomal *tarO* expression on a single-copy plasmid pLI50. Meanwhile, the wild-type strain and the *tarO*-deletion strain carrying the empty plasmid were used as controls. WTAs were extracted from these strains and analyzed by PAGE, showing expected biosynthesis levels (Fig. 2A). The six strains exhibited the same growth pattern and colony morphology on TSB agar plates in the absence of the food preservatives (Fig. 2B). When spotted on agar plates containing various concentrations of TP, SD, or PL, Δ*tarO* cells were observed to present higher susceptibility to TP and SD yet lower sensitivity to PL compared with the wild-type strain (Fig. 2C through H), which is in agreement with the MIC tests. The complementation of *tarO* reverted the cells back to the wild-type phenotype, whereas increased *tarO* expression resulted in a level of compound sensitivity comparable to that of the wild-type strain. In addition, the presence of the empty plasmid, either in the wild type or Δ*tarO* background, did not change the phenotype of the corresponding strains.

Furthermore, the recombinant strains were grown in the presence of various concentrations of TP, SD, or PL for 24 h in liquid media, and the final cell viability was determined using the plating assay. After TP or SD treatment, the lowest viability among all the strains was recorded for the Δ*tarO* cells (Fig. 2I and J), which, however, exhibited the highest viability in the PL challenge (Fig. 2K). Similar growth-inhibiting activity was observed toward the wild-type strain, the *tarO* complementation strain, and the strain with increased *tarO* expression for each preservative. In addition, TP exhibited slight bactericidal activity toward all the strains at their respective MIC values. Collectively, these results suggest that cell susceptibility to the selected food preservatives is closely related to the presence of WTAs.

### Food preservatives change the surface morphology of *S. aureus* in a WTA-dependent manner

**Fig 3:**
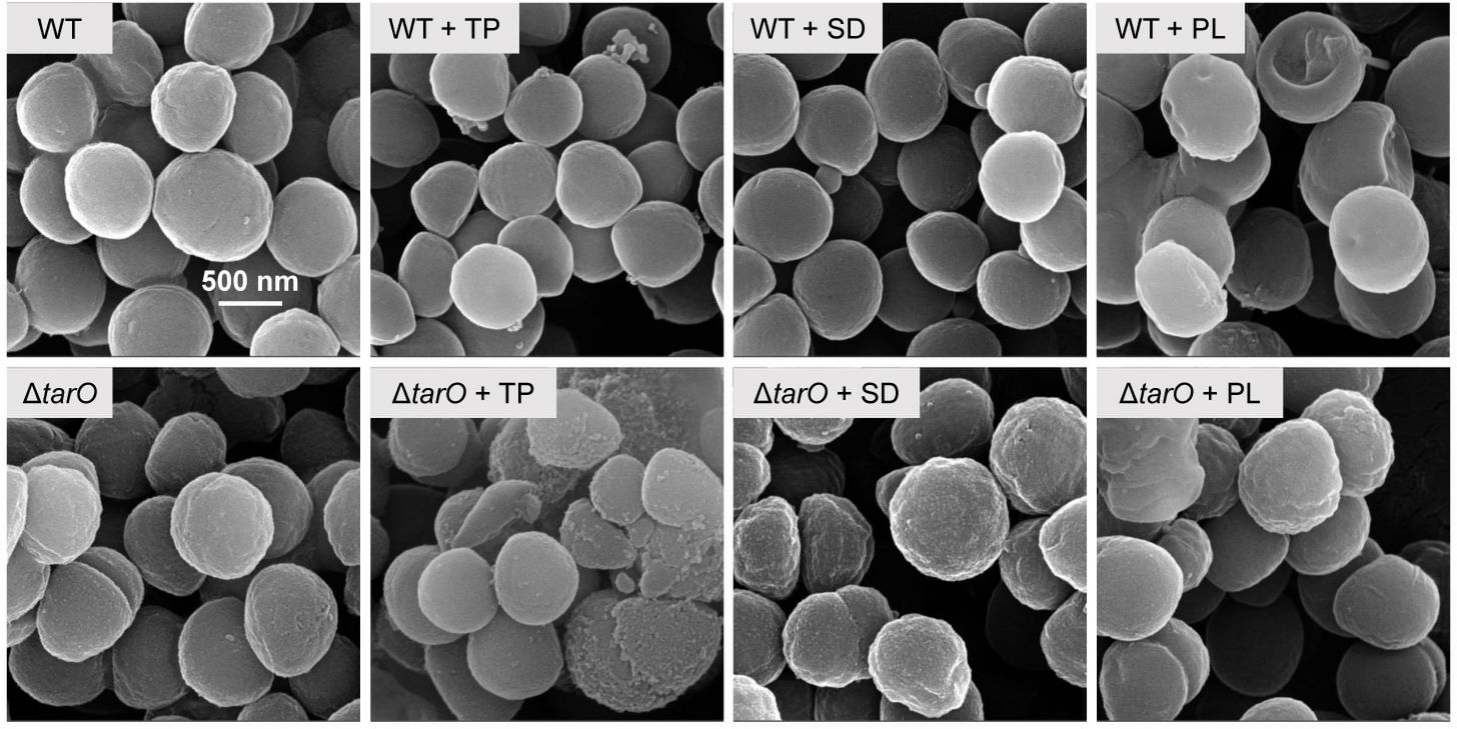
SEM observation of cell surface morphology of *S. aureus* ATCC 6538 WT and Δ*tarO* strains before and after 3 h treatment with 30 µg/mL TP, 4 mg/mL SD, and 0.5 mg/mL PL. These images are representatives of three biological replicates.

Scanning electron microscopy (SEM) was performed to investigate the effects of SD, TP, and PL on cell surface morphology and their potential correlation with the *tarO* gene background (Fig. 3). The wild-type cells appeared as smooth spheres, while deletion of *tarO* alone slightly resulted in the formation of irregular wrinkles and ridges on the cell surface, which is consistent with previous findings (37). Treatment with a sub-MIC of TP or SD did not apparently alter the surface morphology of the wild-type cells, whereas, for the Δ*tarO* cells, cell fracture and more ridges/wrinkles were observed after TP and SD treatment, respectively. Interestingly, PL treatment led to dent formation on the cell surface and inter-connection of individual cells for the wild-type strain, probably owing to defects in separation after cell division. Such defects were remediated upon *tarO* deletion, but the cell surface became rugged. These results are consistent with the sensitivity tests and further demonstrate that the damage caused by the food preservatives to cell structure is closely related to the presence of WTAs.

### Food preservatives induce the expression of WTA biosynthetic genes

**Fig 4:**
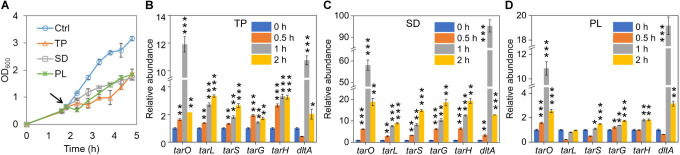
The effect of food preservative treatment on the expression levels of WTA biosynthetic genes. The WT cells of *S. aureus* ATCC 6538 were grown to the mid-log phase and challenged with 0.625 mg/mL TP, 8 mg/mL SD, and 100 mg/mL PL. (**A**) Cell growth after treatment of the preservatives. (**B–D**) Expression levels of *tarO*, *tarL*, *tarS*, *tarG*, *tarH*, and *dltA* were determined by quantitative reverse-transcription PCR using 16s rDNA as an internal reference during a 2 h time course after cell challenge with (**B**) TP, (**C**) SD, and (**D**) PL. The relative gene expression levels in the untreated cells (0 h, right before preservative addition) were used as controls. The black arrow in panel **A** indicates the time of preservative addition. Experiments were performed in biological triplicates. Asterisks indicate statistical significance relevant to the 0 h time point for each gene. *, *P* < 0.05; **, *P* < 0.01; ***, *P* < 0.001.

Given the connection between the WTA and cell sensitivity to the three food preservatives, we wonder whether these antimicrobial compounds could induce gene expression in the WTA biosynthetic pathway. Along this line, we challenged wild-type *S. aureus* ATCC 6538 cells at the mid-log phase with TP, SD, or PL and determined over a 2 h time course the expression levels of the major genes involved in WTA biosynthesis, i.e., *tarO*, *tarL*, *tarS*, *tarG*, *tarH*, and *dltA*. It should be noted that high concentrations of the preservatives were used because cells at the mid-log phase with a relatively high optical density were much less sensitive to these compounds compared with those used in the MIC tests (Fig. 4A). After 1 h treatment with any of the preservatives, *tarO* and *dltA* were up-regulated to extremely high levels, which then dropped dramatically after further treatment to levels that were 1.0- to 17.8-fold higher than those in the untreated cells (Fig. 4B through D). Most of the other genes also showed considerably up-regulated expression upon the preservative challenge. Among these, TP gradually increased the expression of *tarL* and *tarS* in 2 h by 2.3- and 1.6-fold, respectively, whereas the expression of *tarG* and *tarH* appeared to be irrelevant to the exposure time (Fig. 4B). Cell exposure to SD steadily enhanced the expression of *tarL*, *tarS*, *tarG*, and *tarH* by 11.8- to 18.3-fold in a time-dependent manner over 2 h (Fig. 4C). In contrast, PL treatment only induced 48%, 74%, and 82% higher expression of *tarS*, *tarG*, and *tarH*, respectively (Fig. 4D). These results demonstrate that while *tarO* and *dltA* are crucial in self-protection of *S. aureus* from the potential damaging effect of the food preservatives, other genes also contribute to this process yet to distinct extents. In addition, it appears that *tarL*, *tarS*, *tarG*, and *tarH* play minimal roles in cell interaction with PL. It is worth mentioning that PL treatment strongly induced *tarO* expression, which is out of expectation given that *tarO* deletion increased cell survival in PL.

Considering that D-alanylation occurs to WTAs as well as LTAs, another type of surface polymer anchored in the cell membrane that becomes indispensable for cell survival in the absence of WTAs (38), we quantified the expression levels of *ypfP*, *ltaA*, and *ltaS*, which are responsible for initiation, membrane translocation, and chain elongation in LTA biosynthesis, respectively (39). In addition, peptidoglycan as another constituent of the cell wall was also inspected for gene expression related to its biosynthesis, including *mraY*, *murG*, *femX*, and *pbp4* involved in the formation of lipid I precursor, lipid II precursor, pentaglycine bridge, and cell wall crosslinking, respectively (7, 40). Preservative treatment significantly induced the expression of these genes, among which *ltaS*, *pbp4*, and *mraY* were up-regulated by 9.6-fold, 22.4-fold, and 19.5-fold after 1 h treatment with TP, SD, and PL, respectively (Fig. S2).

### WTAs interact with food preservatives

**Fig 5:**
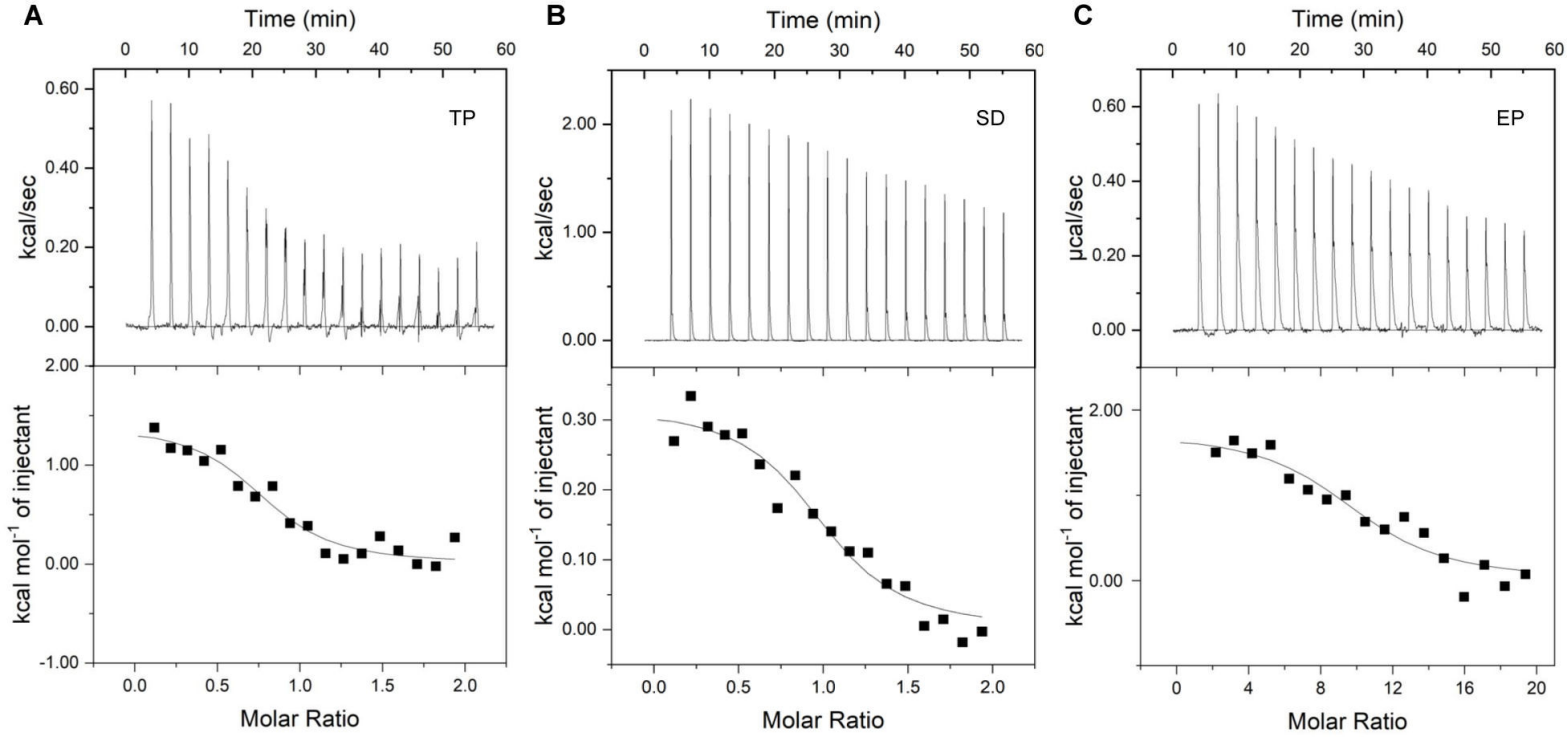
Isothermal titration calorimetry measurement of preservative-WTA interactions. Thermograms (upper row) and the fitted binding isotherms (lower row) are shown for WTA association with (**A**) TP, (**B**) SD, and (**C**) PL.

TP, SD, and PL are small molecules that can theoretically cross the cell wall of *S. aureus*. Preservative-induced expression of WTA biosynthetic genes suggests possible interactions of WTAs with the food preservatives to control their diffusion into the cell. Therefore, we isolated WTAs from wild-type *S. aureus* ATCC 6538 and determined their binding to TP, SD, and PL using ITC (Fig. 5; Table 2). All three compounds could spontaneously bind WTAs with similarly moderate affinity in an endothermic nature, as inferred from the negative ΔG values, the range of the K (binding constant) values, the downward shape of the fitting curves, and the positive ΔH values. The −TΔS exceeds the positive ΔH to result in a negative ΔG, suggesting an entropy-driven binding process. Among the three compounds, TP showed the highest binding affinity to the WTA given the highest K and the lowest ΔG values. In addition, the molar binding ratios between the compounds and WTAs were 1, 1, and 10 for TP, SD, and PL, respectively (Table 2).

**TABLE 2:** Thermodynamic parameters of WTA association with TP, SD, and PL

	Stoichiometry	K (M^−1^)	ΔH (J/mol)	ΔS (J/mol·K)	ΔG (kJ/mol)
TP to WTA	1	7.52 × 10^4^	5882.45	113.04	−28
SD to WTA	1	1.61 × 10^4^	1337.68	84.99	−24
PL to WTA	10	6.81 × 10^4^	7285.03	116.81	−28

**Fig 6:**
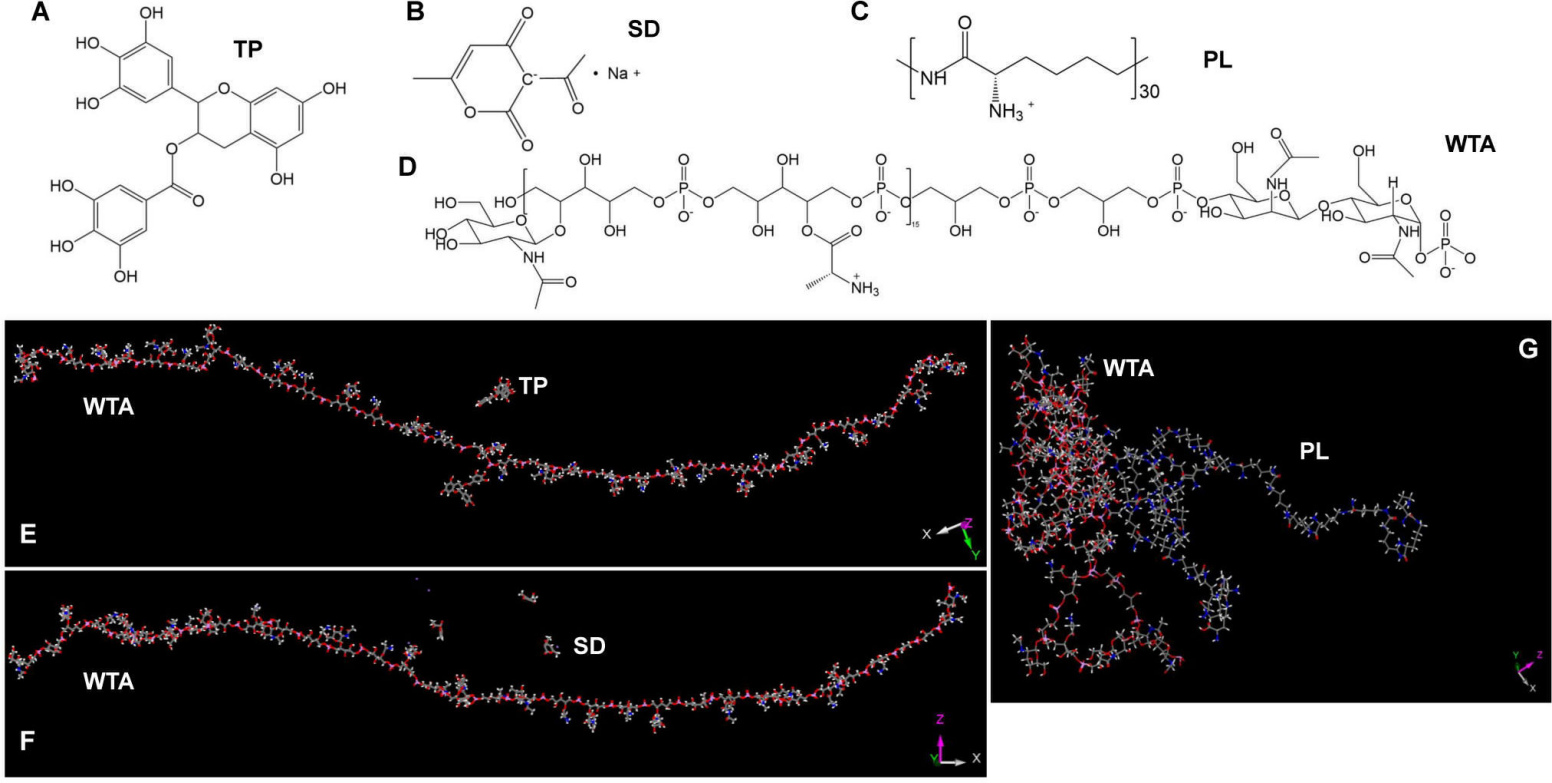
Structures of food preservatives and WTAs for molecular dynamics simulation. (**A–D**) The general structural representations of TP, SD, PL, and WTA for the generation of input structures of the simulation. (**E–G**) The output structures of WTA-TP, WTA-SD, and WTA-PL are at the end of the simulation.

**Fig 7:**
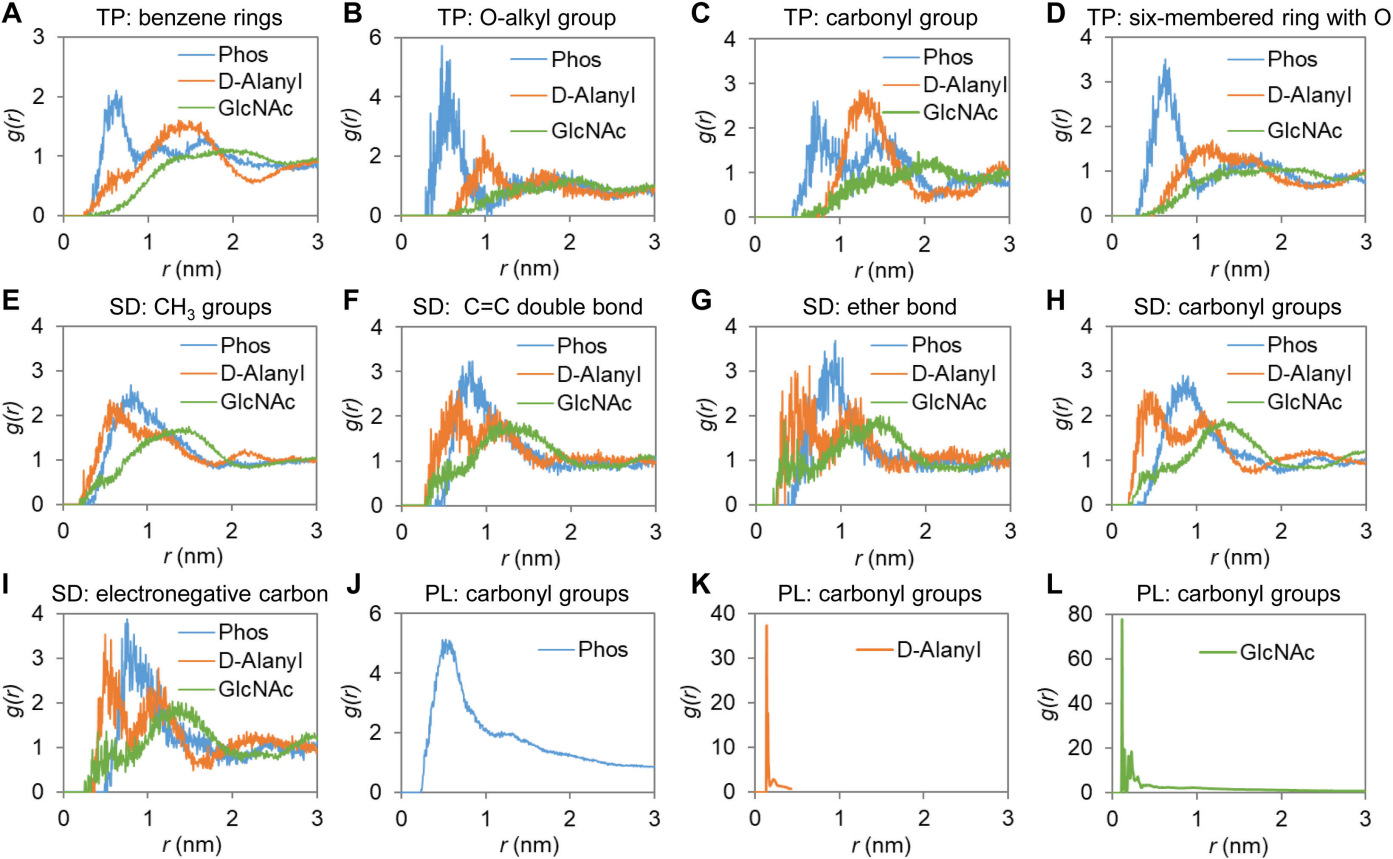
Radial distribution functions of various functional groups of food preservatives relative to the functional groups of WTAs. The local densities of different functional groups of (**A–D**) TP, (**E–I**) SD, and (**J–L**) PL at different radii were analyzed relative to the phosphate group (Phos), D-alanyl ester bond (D-Alanyl), and *N*-acetylglucosamine (GlcNAc) group of the WTA-repeating units.

To gain more details of such interactions, molecular dynamics simulation was performed using structures shown in Fig. 6A through D, with the output structures after simulation shown in Fig. 6E through G. The radial distribution function was employed to evaluate how densely the food preservatives distributed around WTAs within a certain distance, which then serves as an indirect measure of preservative-WTA interactions. As shown in Fig. 7A through D, the benzene ring, the carbonyl group, the O-alkyl group, and the six-membered ring of TP distributed mostly around the phosphate groups and, to a lower extent, around the D-alanyl ester groups, suggesting that TP interacted primarily with the WTA backbone. Since TP is uncharged, whereas oxygen in phosphate groups is electronegative, and D-alanyl ester group is hydrophobic, such interactions may involve hydrogen bonding and hydrophobic interactions. SD mainly gathered around the phosphate groups and D-alanyl ester groups, and various functional groups of SD had almost uniform packing around the WTA, suggesting that SD as a whole accumulated close to the WTA, probably forming charge-charge interactions and hydrogen bonds (Fig. 7E through I). The carbonyl groups of PL accumulated in extremely close proximity to D-alanylation and glycosylation sites (Fig. 7J through L). As the oxygen in carbonyl groups is highly electronegative, and as both PL and D-alanylation sites are positively charged under physiological conditions, the observed PL-WTA interaction was probably hydrogen bonding.

### Cell sensitivity to food preservatives is differentially affected by D-alanylation

**Fig 8:**
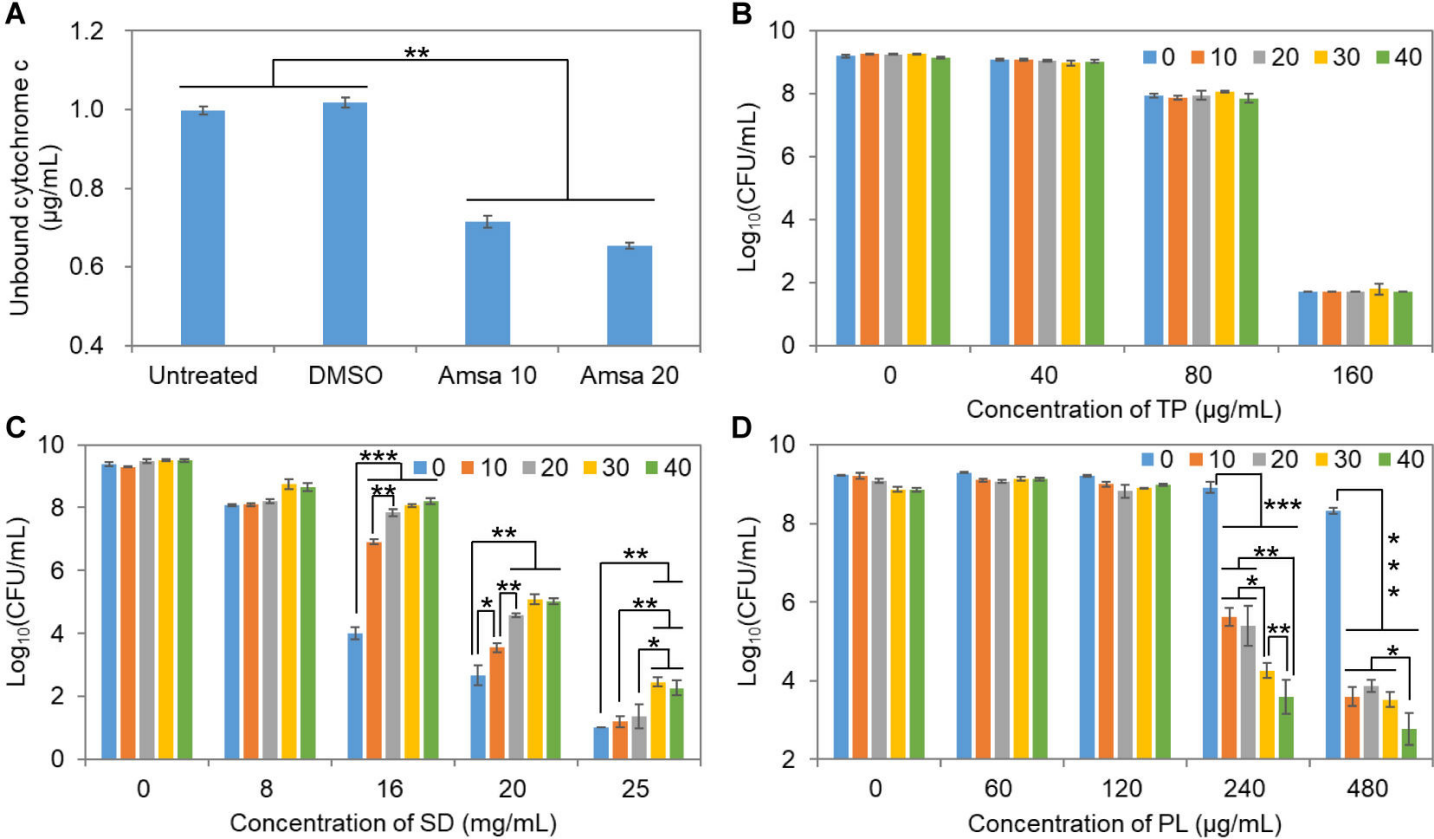
Inhibition of D-alanylation modification has an impact on cell surface charge and cell susceptibility to preservatives. (**A**) Amount of cytochrome c (positively charged) unbound to *S. aureus* cell surface in the absence or presence of amsacrine at 10 or 20 µg/mL. (**B–D**) Cell viability in the presence of TP, SD, or PL after treatment with 0, 10, 20, 30, or 40 µg/mL of amsacrine. Data are presented as mean ± standard deviation of biological triplicates. *, *P* < 0.05; **, *P* < 0.01; ***, *P* < 0.001.

D-alanylation of WTAs as an important tailoring modification protects *S. aureus* from positively charged antimicrobials via charge repulsion. Based on the results that TP, SD, and PL drastically increased the expression level of *dltA*, and considering that SD and PL dissociate to form negatively and positively charged particles in PBS, respectively, it is necessary to investigate how cell susceptibility to these preservatives is affected by the extent of D-alanylation. Toward this goal, we grew *S. aureus* wild-type cells in the presence of amsacrine, a known inhibitor of the D-alanylation modification of teichoic acids (41). Cells were then exposed to cytochrome c, a heavily positively charged protein under physiological conditions that can bind to the negatively charged regions on the cell surface. Amsacrine treatment at 10 or 20 µg/mL decreased the concentration of the unbound cytochrome c by 28% or 35%, respectively, relative to the untreated control, whereas this effect was not observed after DMSO treatment, which was used as the solvent for amsacrine preparation (Fig. 8A). Meanwhile, amsacrine exposure reduced the MIC of gentamicin and neomycin (positively charged antibiotics) both by eight folds (Table S2), demonstrating considerable reduction in the number of positive charges on *S. aureus* cell surface.

When amsacrine-treated cells were challenged with TP for 24 h, cell viability showed no variations compared to the untreated control (Fig. 8B). In contrast, amsacrine significantly increased cell survival in SD (Fig. 8C) but dramatically reduced cell viability in PL in a dose-dependent manner (Fig. 8D). These observations, as expected, suggest that in the absence of D-alanylation and with an increase in the number of net negative charge on the cell surface, Coulomb’s force is critical to cell-preservative interaction.

### WTA glycosylation affects cell susceptibility to food preservatives

**Fig 9:**
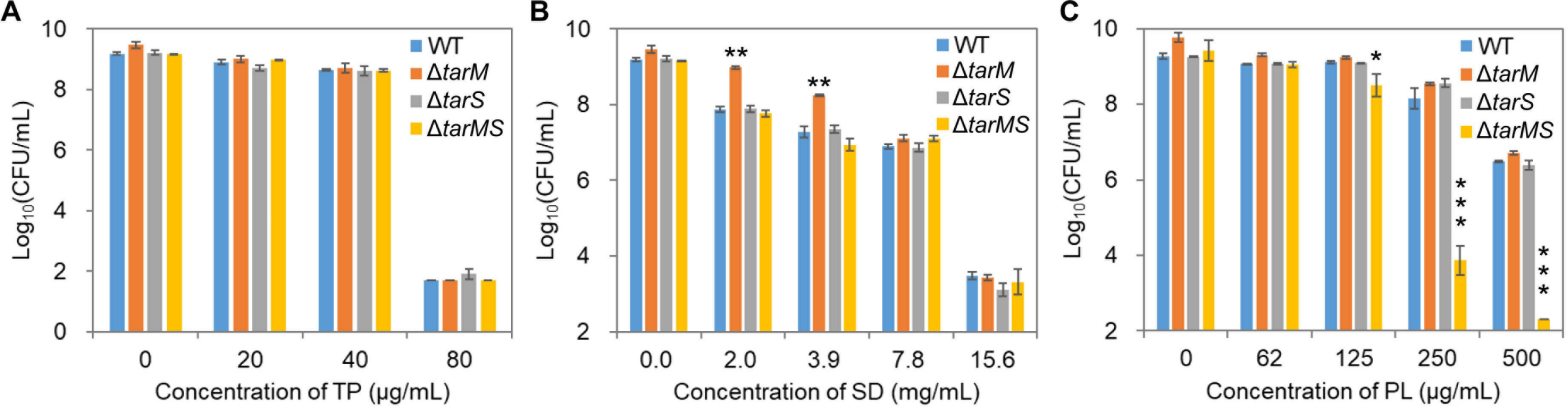
Impact of WTA glycosylation on the susceptibility of *S. aureus* RN4220 to food preservatives. Cells were grown for 24 h in TSB media supplemented with (**A**) TP, (**B**) SD, and (**C**) TP at various concentrations, and cell viability was measured via the plating assay. Δ*tarM*/*S*/*MS*, cells with gene deletion of *tarM*, *tarS*, or *tarMS* from the genome. Data are presented as mean ± standard deviation of biological triplicates. *, *P* < 0.05; **, *P* < 0.01; ***, *P* < 0.001.

Glycosylation of *S. aureus* WTAs is crucial to cell resistance to antimicrobials. Therefore, we further investigated the role played by WTA glycosylation in mediating cell susceptibility to food preservatives. Given that *S. aureus* ATCC 6538 only has β-glycosylation due to the absence of *tarM* in its genome, we adopted strain RN4220, which shares the same WTA biosynthetic pathway as ATCC 6538 but contains both α- and β-glycosylation catalyzed by TarM and TarS, respectively (36, 42, 43). The wild-type strain RN4220 was more sensitive to TP and PL than strain ATCC 6538, as lower MICs were observed (Table S3). Upon single or double deletion of the *tarMS* genes, there was no discrimination of cell sensitivity to TP (Fig. 9A), which is consistent with the simulation result that TP had no apparent interactions with the glycosyl units. SD at 2.0 and 3.9 mg/mL was significantly less sensitive toward the Δ*tarM* strain, which showed one-order-of-magnitude higher cell viability after 24 h of SD treatment compared with other strains (Fig. 9B). However, such a difference decreased at a higher SD concentration. In addition, it is interesting to find that the Δ*tarM* phenotype was not carried by the Δ*tarMS* strain. When cells were treated with PL at 250 and 500 µg/mL, the Δ*tarMS* strain was significantly more sensitive, with the final cell counts differing by more than four orders of magnitude in comparison with other strains (Fig. 9C).

## DISCUSSION

In the current work, we investigated the effect of WTAs in mediating *S. aureus* susceptibility to commonly used antimicrobial food preservatives and identified three compounds, i.e., TP, SD, and PL, to which the cell sensitivity was closely correlated with the presence of WTAs. Using a series of assays combined with molecular dynamics simulation, we revealed that these preservatives stimulated the expression of WTA biosynthetic genes, spontaneously interacted with WTA backbone and modification sites with moderate affinity, and responded differentially to the pattern of WTA glycosylation and D-alanylation. Our observations indicate that multiple mechanisms related to WTAs are utilized by *S. aureus* for self-protection against these antimicrobial compounds.

TP exhibits antimicrobial effects via disruption of the cell wall and cell membrane, inhibition of lipid synthesis, and generation of oxidative stress (44–46). Therefore, control over its passage into the cell is essential for cell survival. As WTAs were observed to bind TP with moderate affinity spontaneously via predicted hydrogen bonding and hydrophobic interactions, it is no wonder that this preservative can be trapped in the WTA layer to reduce its entry to the cytoplasm. The facts that cell exposure to TP induced *tarO* expression and that cells with *tarO* deletion became more vulnerable provide further evidence that WTAs are exploited as a countermeasure against the potential damage caused by TP, which is in line with a previous finding that TP represses the expression of *glpQ* in *S. aureus* (47) to reduce WTA degradation (48, 49). The up-regulation of LTA and peptidoglycan biosynthesis further enhances the robustness of the cell wall. It is worth noting that TP induced up-regulation of *tarS* and *dltA* expression despite a lack of correlation between WTA modification and cell susceptibility to TP. It is likely that the oxidative stress generated by TP induced the expression of the two-component system GraRS (50), which then positively regulates transcription factors Agr and MgrA (51) to up-regulate the expression of *tarH* and *dlt* genes, respectively (52, 53). This results in more display of WTAs on the cell surface with more positive charges. In addition, the simulation predicted binding between the WTA backbone and D-alanylation sites with TP. Therefore, the benefits of having more D-alanylation on WTAs are probably retainment of TP in the cell wall via hydrogen bonding, whereas the glycosyl units may serve as a modulator of cell wall compactness and permeability, as observed in *L. monocytogenes* that uses WTA glycosylation to reduce the permeability of the cell wall and to resist cationic antimicrobial peptides (54, 55).

SD is widely used in food and pharmaceutical industries. This compound disrupts bacterial cell membranes, induces the glyoxylate pathway, and inhibits cell respiration (56). In *S. aureus*, respiration generates a proton gradient on the cell surface that is maintained and stabilized by WTAs (57, 58). With SD treatment, the reduced respiration and the dissipated membrane potential are associated with a lower local proton concentration, which can then be compensated for by up-regulation in WTA/LTA biosynthesis and D-alanylation. Although a direct outcome of this process is a decline in the net negative charge on the cell surface and more binding of SD, such binding helps to guide SD away from the membrane and indirectly facilitates cell survival. With regard to the impact of glycosylation, it is surprising to observe lower cell susceptibility to SD upon *tarM* knockout but not *tarMS* deletion. It is possible that WTA α-glycosylation catalyzed by TarM generates special steric hindrance for SD-WTA interaction, while *tarM* deletion favors SD binding and trapping in the cell wall layer; in the absence of both TarM and TarS, however, the relieved steric hindrance is compromised by higher permeability of the cell wall due to a lack of both types of glycosylation. Nonetheless, it is still unclear why the effect of *tarM* was only apparent at moderate SD concentrations. Generally, *tarS* in *S. aureus* contributes to cell resistance to antibiotics due to unknown reasons (12), and *tarP* in certain prophage-carrying strains guides resistance to antibiotics and phages (14). Our observation uncovers that *tarM* is also potentially involved in drug tolerance, and more exploration is required to obtain a clear understanding of its specific function.

PL is a cationic antimicrobial peptide, and its antimicrobial mechanism against *S. aureus* is peptidoglycan damage and membrane depolarization followed by PL entry into the cell to induce the generation of reactive oxygen species, inhibit central carbon metabolism, and interfere with DNA replication and transcription (59, 60). The D-alanylation modification of WTAs incorporates local positive charges to the negatively charged cell surface, thereby protecting cells from the damaging effect of PL via charge repulsion. Indeed, cells exposed to PL strongly up-regulated the expression of *dltA*, and inhibition of D-alanylation by amsacrine made the cell surface more negatively charged (41) and reduced cell survival in PL. Similar to D-alanylation, a lack of glycosylation of WTAs via *tarMS* deletion resulted in higher sensitivity to PL compared with the wild-type phenotype, and PL treatment slightly enhanced *tarS* expression. Based on the ITC and modeling results, it is likely that the interaction between the glycosyl units and PL sterically hinders PL binding to the negatively charged regions distributed on the cell surface, thus reducing its contact with the cell membrane.

Theoretically, the Δ*tarO* strain without WTAs and the glycosylation/D-alanylation modifications should be more sensitive to PL. However, the opposite result was observed, probably due to two reasons. First, *dltA* is mostly involved in D-alanylation of LTAs, which then transfer the D-alanyl groups to WTAs (61). Moreover, LTAs and their D-alanylation modification become essential for the survival of cells lacking WTAs (38), and the level of LTAs is inversely correlated with that of WTAs (62). Therefore, the observed induction of *dltA* and LTA biosynthetic genes by PL indicates that more LTAs are synthesized and D-alanylated to repulse PL in compensation for *tarO* deletion. Second, despite the defects in the Δ*tarO* cell wall (12, 63), the up-regulation of LTA and peptidoglycan biosynthesis helps to stabilize the cell wall (63), physically restricting the access of PL to the cell membrane.

Food preservatives are usually adopted at different concentrations depending on the type of food, and the maximum permissible concentrations in food for TP, SD, and PL are 0.4 g/kg, 1 g/kg, and 5 g/kg, respectively (45, 64). The MICs of TP and PL in the current study are within these ranges, while the MIC of SD exceeds the allowed dose. SD as a preservative is always used in bakery food and pickled vegetables, the former being low in water content whereas the latter is low in nutrients in an acidic environment. Therefore, a low SD concentration is sufficient to inhibit microbial growth. In the current study, *S. aureus* was grown in TSB, which is a rich medium to support optimal cell growth, thus becoming more robust and requiring a higher SD concentration to achieve growth inhibition. The impact of WTAs on cell vulnerability to these food preservatives indicates that it is important to periodically evaluate the efficacy of these compounds against common isolates of *S. aureus* found in the food industry and adjust the doses or adopt multiple preservatives in combination when necessary.

In summary, we have demonstrated that WTAs are closely related to *S. aureus* susceptibility to three antimicrobial food preservatives via distinct mechanisms, which include (i) charge repulsion mediated by D-alanylation to reduce the packing of the antimicrobials around the cells, (ii) hydrogen bonding and hydrophobic interactions to maintain the antimicrobial compounds in the WTA layer and to reduce their passage across the cell membrane; (iii) steric hindrance to minimize the contact of antimicrobials with the cell membrane. Taking into consideration the prevalence of *S. aureus* in raw and packaged food and its fast development of resistance to antimicrobials, our work raises the possibility and concern that *S. aureus* in food may develop resistance to preservatives by adopting WTA-related machineries and thus calls for more stringent control to guarantee food safety and public health.

## SUPPLEMENTAL MATERIAL

Supplemental material aem.01023-25-s0001.pdf Tables S1 to S3; Figures S1 and S2.

ASM does not own the copyrights to Supplemental Material that may be linked to, or accessed through, an article. The authors have granted ASM a non-exclusive, world-wide license to publish the Supplemental Material files. Please contact the corresponding author directly for reuse.
