# Supplementary material for: Mechanistic study on the susceptibility of Staphylococcus aureus to common antimicrobial preservatives mediated by wall teichoic acids
Source: Appl Environ Microbiol. 2025 Jun 30;91(7):e01023-25. doi: 10.1128/aem.01023-25 (PMC12285261; doi:10.1128/aem.01023-25)
Supplement: Supplemental material — Tables S1 to S3; Figures S1 and S2. [file aem.01023-25-s0001.pdf]

SUPPLEMENTARY INFORMATION

Mechanistic Study on the Susceptibility of *Staphylococcus aureus* to Common Antimicrobial Preservatives Mediated by Wall Teichoic Acids

Xia Wu[1,2]*, Jiayi Wang[1,2], Ji Li[3], Zheng Su[1,2], Jian Zha[1,2]*

[1]School of Food Science and Engineering, School of Biological and Pharmaceutical Sciences, Shaanxi University of Science and Technology, Xi'an, Shaanxi 710021, China

[2]Xi'an Key Laboratory of Antiviral and Antimicrobial-Resistant Bacteria Therapeutics Research, Xi'an, Shaanxi 710021, China

[3]School of Bioresources and Materials Engineering, Shaanxi University of Science and Technology, Xi'an, Shaanxi 710021, China

*Correspondence:

Xia Wu: wuxia@sust.edu.cn;

Jian Zha: zhajian1985@sust.edu.cn

Table S1. List of primers used in this study

Name	Sequence (5' to 3')
**Gene expression and complementation**	
tarO-F	CGCGGATCCATGGTTACATTATTACTAGT
tarO-R	ACGCGTCGACCTATTCCTCTTTGTG
**Gene knockout**	
tarM-up-R	ATACCTAGGATTTTTTCATTATAATTATTCCTTTACCTCA
tarM-down-F	GGTAAAGGAATAATTATAATGAAAAAATCCTAGGTATTCA
tarM-19-F	TGTAAAACGACGGCCAGTGACAGATGATTATTCAATCATTA
tarM-19-R	CTATGACCATGATTACGCCTTACCTCCAAACAACTTTAGTAG
pKOR1-tarM-F	GTTGTTTGGAGGTAACCTGTCGTGCCAGCTGCATTAATGA
pKOR1-tarM-R	ATTGAATAATCATCTGTCGGTACCGGTTCCGAGGCTCAAC
tarM-pKOR1-F	GCCTCGGAACCGGTACCGACAGATGATTATTCAATCATTA
tarM-pKOR1-R	TGCAGCTGGCACGACAGGTTACCTCCAAACAACTTTAGTA
tarS-19-F	TGTAAAACGACGGCCAGTTGGGAAGGCAGACTACATCTTT
tarS-19-R	CTATGACCATGATTACGCCCGAAAAACTGCTGGACTTATT
tarS-up-R	TGTTGGCTCACAATGATTTGAGGGCATTTATATACCTCTC
tarS-down-F	TATATAGTAGTCAAAGTGGGAGAGGTATATAAATGCCCTC
pKOR1-tarS-F	AAGTCCAGCAGTTTTTCGCCTGTCGTGCCAGCTGCATTAA
pKOR1-tarS-R	ATGTAGTCTGCCTTCCCAGGTACCGGTTCCGAGGCTCAACGT
tarS-pKOR1-F	GCCTCGGAACCGGTACCTGGGAAGGCAGACTACATCTTT
tarS-pKOR1-R	ATGCAGCTGGCACGACAGGCGAAAAACTGCTGGACTTATT
**qRT-PCR**	
q-16SrDNA-F	ACGTGGATAACCTACCTATAAGACTGGGAT
q-16SrDNA-R	TACCTTACCAACTAGCTAATGCAGCG
q-tarO-F	TGGATTGGTCATCCTATTGAAACAG
q-tarO-R	CTAAGGCAGCGGCAATTTGA
q-tarL-F	TAGATGGGAATCAATTCGTTTGTCG
q-tarL-R	TCACATCTGGGTTTAACTGTGCAAT
q-tarS-F	GGTCCAGGTAAACCTAGAAATGTGG
q-tarS-R	CATCAATAAATGCTGCTGCATCCTT
q-tarG-F	ATTCAAAGACTGGCTCAGTTTCAAG
q-tarG-R	CCTAATCCAAAAACCATCCAGTAAACC
q-tarH-F	TAAAGAACGTATGAAAGATGCGCTC
q-tarH-R	TGATGCCAACAAGCCCTATGAC
q-dltA-F	GCGTTAGTAAACCGTTTCCC
q-dltA-R	ACCTTCGATAACAAGTTCACC
q-ypfP-F	AGCGAAAAGTGCAAATGCACAAG
q-ypfP-R	TGGAATACAACGGGCGAAACC
q-ltaA-F	AAATTGTTGATGTGACAAAGCGCC
q-ltaA-R	TCGAAACTGCACAGCCGATACC
q-ltaS-F	TGGCGGCTTCAGTAGCATTATTC
q-ltaS-R	GTTAGGCTCTGTTTGACGTTGTTTC
q-mraY-F	AGGTCCACAAAGCCATATGAAGAAG
q-mraY-R	GCAATACCAATTTGCGCCAAAAAC
q-murG-F	GAGCTGGATCAAATGCGATTTATG
q-murG-R	TCGAGTTCTTTCCTGTTCCATTTC
q-femX-F	GGGAACGCGATGGCTTCTTAAC
q-femX-R	TGCGCTTTTTTAGCTTGCTTTTCAG
q-pbp4-F	CTGCTAACAGTGACGTAACCCC
q-pbp4-R	GCTGCCCCTTATTTACAGCTTCC

Table S2. MICs of gentamycin and neomycin against *S. aureus* ATCC 6538 in the absence and presence of amsacrine

	Gentamycin (µg/mL)	Neomycin (µg/mL)
No amsacrine	160	400
With amsacrine (20 µg/mL)	20	50

Table S3. MICs of TP, SD, and PL against *S. aureus* strain RN4220.

Compound	TP	SD	PL
MIC	80 µg/mL	15.6 mg/mL	560 µg/mL

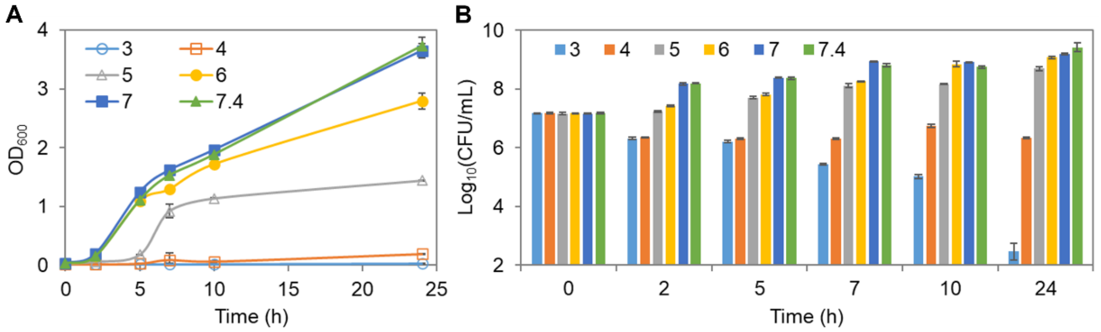

Figure S1. Growth of *S. aureus* ATCC 6538 under various pH conditions. (A) Culture turbidity; (B) cell viability.

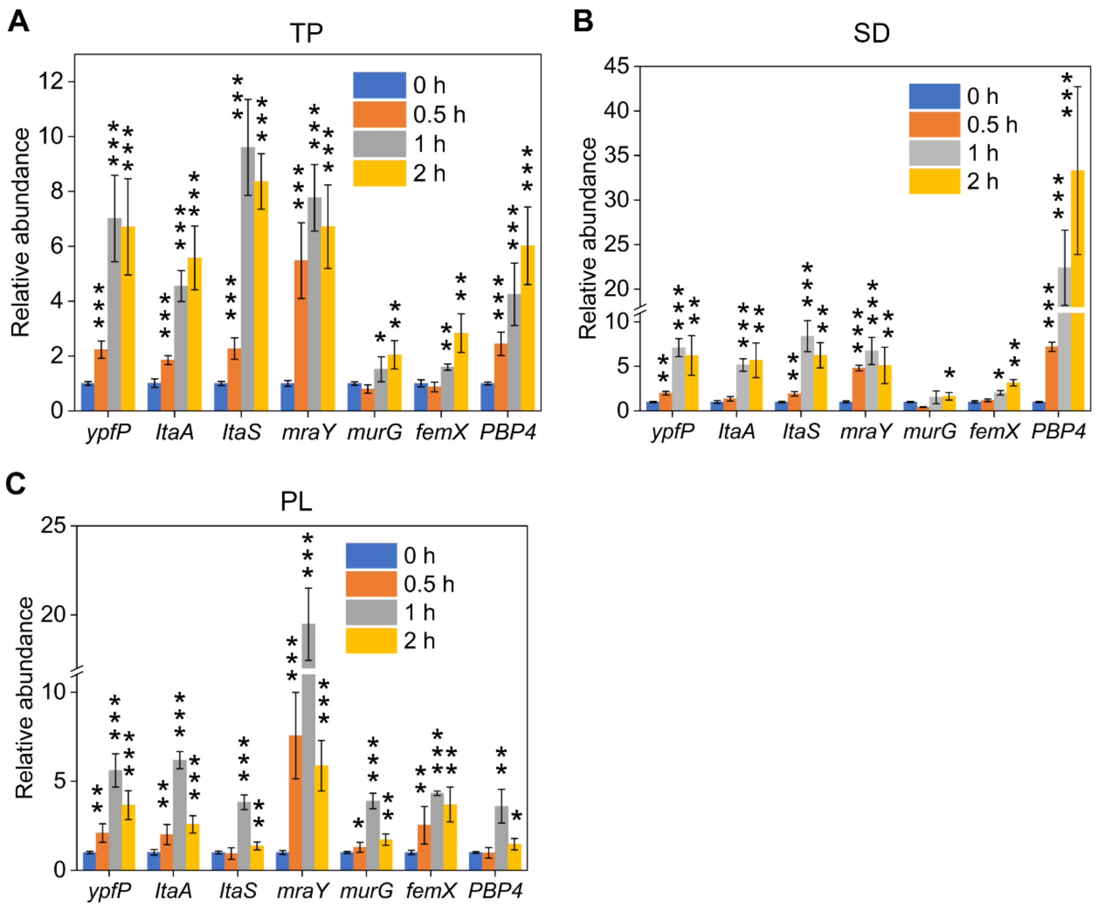

Figure S2. The effect of food preservative treatment on the expression levels of LTA and peptidoglycan biosynthetic genes. The wildtype cells of *S. aureus* ATCC 6538 were grown to the mid-log phase and challenged with 0.625 mg/mL TP, 8 mg/mL SD, and 100 mg/mL PL. Expression levels of *ypfP*, *ltaA*, *ltaS*, *mraY*, *murG*, *femX* and *pbp4* were determined by qRT-PCR using 16s rDNA as an internal reference during a 2 h time course after cell challenge with (A) TP, (B) SD, and (C) PL. The relative gene expression levels in the untreated cells (0 h, right before preservative addition) were used as controls. Experiments were performed with four biological replicates. Asterisks indicate statistical significance relevant to the 0 h time point for each gene. *, $P<0.05$; **, $P<0.01$; ***, $P<0.001$.
